# Configural relations in humans and deep convolutional neural networks

**DOI:** 10.3389/frai.2022.961595

**Published:** 2023-03-01

**Authors:** Nicholas Baker, Patrick Garrigan, Austin Phillips, Philip J. Kellman

**Affiliations:** ^1^Department of Psychology, Loyola University Chicago, Chicago, IL, United States; ^2^Department of Psychology, Saint Joseph's University, Philadelphia, PA, United States; ^3^UCLA Human Perception Laboratory, Department of Psychology, University of California, Los Angeles, Los Angeles, CA, United States

**Keywords:** perception of relations, deep convolutional neural networks, DCNNs, deep learning, abstract relations, visual relations, shape perception, abstract representation

## Abstract

Deep convolutional neural networks (DCNNs) have attracted considerable interest as useful devices and as possible windows into understanding perception and cognition in biological systems. In earlier work, we showed that DCNNs differ dramatically from human perceivers in that they have no sensitivity to global object shape. Here, we investigated whether those findings are symptomatic of broader limitations of DCNNs regarding the use of relations. We tested learning and generalization of DCNNs (AlexNet and ResNet-50) for several relations involving objects. One involved classifying two shapes in an otherwise empty field as same or different. Another involved enclosure. Every display contained a closed figure among contour noise fragments and one dot; correct responding depended on whether the dot was inside or outside the figure. The third relation we tested involved a classification that depended on which of two polygons had more sides. One polygon always contained a dot, and correct classification of each display depended on whether the polygon with the dot had a greater number of sides. We used DCNNs that had been trained on the ImageNet database, and we used both restricted and unrestricted transfer learning (connection weights at all layers could change with training). For the same-different experiment, there was little restricted transfer learning (82.2%). Generalization tests showed near chance performance for new shapes. Results for enclosure were at chance for restricted transfer learning and somewhat better for unrestricted (74%). Generalization with two new kinds of shapes showed reduced but above-chance performance (≈66%). Follow-up studies indicated that the networks did not access the enclosure relation in their responses. For the relation of more or fewer sides of polygons, DCNNs showed successful learning with polygons having 3–5 sides under unrestricted transfer learning, but showed chance performance in generalization tests with polygons having 6–10 sides. Experiments with human observers showed learning from relatively few examples of all of the relations tested and complete generalization of relational learning to new stimuli. These results using several different relations suggest that DCNNs have crucial limitations that derive from their lack of computations involving abstraction and relational processing of the sort that are fundamental in human perception.

## 1. Introduction

The perception of objects, spatial layouts, and events are crucial tasks of intelligent systems, both biological and artificial. For these tasks, information in reflected light affords the richest information. Differences in material substances' absorption and reflection of light carry information about boundaries and shapes of objects and surfaces, as well as their spatial location and relations, textures, and material properties. The concentration of research effort on vision in human and artificial systems is no accident, given the detailed information available in reflected light, its spatial and temporal precision, and its availability at a considerable distance from objects and events themselves.

In human vision, research has identified specialized processes and neural mechanisms that contribute to visual perception and representation of objects, spatial layout, motion, and events. Among these are processes that separate figure from ground and determine border ownership (Rubin, 1915/1958; Koffka, 1935; Driver and Baylis, 1996; Zhou et al., 2000), detect complete objects despite fragmentation due to occlusion or camouflage (Michotte et al., 1964; Kanizsa, 1979; Kellman and Shipley, 1991; Kellman and Fuchser, in press), represent the shapes of contours, objects, and surfaces (Wallach and O'Connell, 1953; Ullman, 1979; Marr, 1982; Biederman, 1987; Lloyd-Jones and Luckhurst, 2002; Pizlo, 2008; Elder and Velisavljević, 2009; Baker and Kellman, 2021), determine the direction of motion (Adelson and Movshon, 1982), and use relational information to perceive events (Michotte, 1954; Johansson, 1978). All of these processes appear to involve computational processes and dedicated neural machinery specialized to extract and represent important structural properties of scenes and events.

A consistent hallmark of these and other aspects of human visual processing is the importance of relations. Relations are crucially involved in visual perception in two related but separable ways. First, capturing important properties of the world involves relational information in the optical input and perceptual mechanisms that can extract it. Relevant relations as stimuli for vision often involve considerable complexity (Johansson, 1978; Gibson, 1979; Ullman, 1979; Marr, 1982; Palmer et al., 2006; Baker and Kellman, 2018). Second, the outputs of perception involve explicit representations of relational properties—relations across space, such as shape or arrangement (Koffka, 1935; Baker and Kellman, 2018), or properties based on patterns across time, such as causality or social intention (Heider and Simmel, 1944; Michotte, 1954; Scholl and Tremoulet, 2000). Evidence indicates the abstract nature of these and other perceptual representations (e.g., Izard et al., 2009; Hummel, 2011; Baker and Kellman, 2018). The representation of relational properties in the output allows perceptual descriptions to subserve a wide variety of tasks and to connect naturally to thought, action, and learning (Gibson, 1969; Garrigan and Kellman, 2008; Klatzky et al., 2008; Kellman and Massey, 2013).

Efforts in artificial vision have sought to develop algorithms for extraction of information that might produce explicit representations of contours, surfaces, spatial layout, objects, and shape (Marr, 1982). For object recognition, these efforts have led to proposals for solving the relevant computational tasks explicitly using information about shape (Bergevin and Levine, 1993; Belongie et al., 2002; Pizlo, 2008; Rezanejad and Siddiqi, 2013), local texture patterns (Lowe, 1999), or surface feature segmentation (Shi and Malik, 2000; Shotton et al., 2009).

Although these efforts have yielded important progress, they have been overshadowed in recent years by results from a wholly different approach: deep convolutional neural networks (DCNNs). DCNN architectures have many applications, but one clear focus, and area of conspicuous success, is in image classification. In DCNNs, object recognition is not based on explicitly encoded contours, surfaces, or shapes of objects present in images (Krizhevsky et al., 2012). Instead, the networks learn to accurately classify many images depicting various object categories from the weighted combination of the responses of many small, local filters, the responses of which are themselves learned.

The successes of deep networks in object recognition have led to research questions flowing in the opposite direction from many earlier efforts. Rather than starting with biological vision phenomena, such as segmentation of figure from ground or completion of partly occluded objects, and attempting to construct computer vision models to perform these tasks, many researchers are currently investigating similarities between deep networks trained for object recognition and the human visual system. Node activity in intermediate layers of deep networks correlates with activity of cell populations in V4 (Pospisil et al., 2018) and some deep networks have been found to be predictive of cell populations in IT (Yamins et al., 2014). Deep networks trained for object recognition also appear to predict human behavior in judging the similarity between objects (Peterson et al., 2016), the memorability of objects (Dubey et al., 2015), and the saliency of regions in an image (Kümmerer et al., 2014).

At the same time, other research has suggested that deep learning approaches have deep limitations. These limitations are being studied in terms of the applicability of deep learning systems as models of biological processing but also regarding their impact in applications to consequential real-world tasks. Ultimately, such inquiries may help to determine both the ways in which the characteristics of deep learning networks are embodied in aspects of biological vision and ways in which deep learning approaches can be enhanced by incorporating specialized adaptations that are evident in biological systems.

In earlier work, we reported that DCNNs that successfully classify objects differ from human perceivers in their access to and use of shape (Baker et al., 2018). Kubilius et al. (2016) had tested shape as a cue for recognition and found that DCNNs can classify silhouettes with about 40% accuracy and showed sensitivity to non-accidental features of objects [e.g., parallel vs. converging edges (Biederman, 1987)]. In our research, we showed that DCNNs showed a clear lack of sensitivity to global shape information. This conclusion rested on multiple, converging tests. When texture and shape conflicted (as in a teapot with golf ball texture), the networks classified based on texture; glass ornaments readily recognizable by humans as animals or objects were poorly classified by DCNNs; DCNNs showed poor performance in classifying silhouettes of animals, and they showed no ability to correctly classify outline shapes (Baker et al., 2018). Examining error patterns led us to suggest a distinction between local contour features and more global shape. DCNNs clearly access the former but seem to have no access to the latter. We tested this hypothesis with silhouettes of objects that DCNNs had correctly classified, altered in two different ways in separate experiments. In one, we scrambled the spatial relations between object parts to destroy their global shape features while preserving many of the local edge properties present in the original stimulus. In the second, we preserved global shape but altered local edge features by adding serrations to the bounding contours of objects. Although human recognition of part-scrambled objects was highly disrupted, DCNN responses were little affected by scrambling. In contrast, the use of local serrated edges to define overall shape had little effect on human classifications but completely disrupted the network's classification of objects (Baker et al., 2018).

Subsequent work provided further evidence that DCNNs have little or no sensitivity to global shape. Baker et al. (2020b) found that networks they trained to discriminate squares and circles would consistently classify as circles squares whose edges were comprised of concatenations of curved elements. Similarly, circular patterns made from concatenations of small corner elements were classified as squares. These results were relatively consistent across a variety of DCNNs (AlexNet, VGG-19, and ResNet-50), and for both restricted and unrestricted transfer learning (Baker et al., 2020b).

These and other results pose clear contrasts with research on human visual perception, in which shape is the primary determinant of object recognition (Biederman and Ju, 1988; Lloyd-Jones and Luckhurst, 2002; Elder and Velisavljević, 2009). Shape is represented even when it must be abstracted from disconnected stimulus elements (Baker and Kellman, 2018). In fact, the specific, directly accessible local features from which shape is extracted are often not encoded in any durable representation (Baker and Kellman, 2018) and may in many cases be represented as statistical summaries rather than precise records of features in particular positions (Baker and Kellman, in press).

### 1.1. Motivation of the present research

It might be natural to interpret the limitations of DCNNs with regard to global shape as deriving from the absence in these networks of specialized shape extraction and representational processes that have evolved and proven useful in human vision. Although we believe aspects of that point of view are likely correct, we wondered whether the limitations in capturing shape relations in DCNNs might be indicative of a more general limitation regarding relations.

A basic reason for supposing that DCNNs might have a general limitation with regard to relations involves the convolution operation at the heart of much of DCNN processing. Convolution applied to an image input is inherently a local process and a literal process. The output of a convolution operator at the location of its center is the weighted sum of image values of intensity in a neighborhood of locations around the center. At later layers, convolution may be applied to the values obtained by a prior convolution operation or some kind of pooling operation, such as max pooling, which reduces the size of the array by assigning to larger neighborhoods the maximum value of operator outputs in that region. There is little doubt that these operations have high utility and flexibility. The convolutional kernels that develop through learning can assume a vast variety of forms. Likewise, one or more fully connected layers in a DCNN can allow the development, through changes of weights in training, of sensitivity to a wide variety of relations between even spatially separated locations. DCNNs can theoretically capture an enormous number of potential relations in images, many of which would defy easy verbal description by humans and would never be designed in a priori attempts to capture important properties.

Yet not all relations are created equal. There may still be important limitations regarding most DCNNs and relations. In particular, relations that require explicit representation or abstraction may be problematic. This idea would fit with previously discovered limitations regarding shape. As emphasized in classic work by Gestalt psychologists (e.g., Koffka, 1935), shape is an abstract relational notion. A square may be made of small green dots in particular locations, but neither relations defined over green dots nor specific locations are intrinsic to the idea of squareness. Any tokens will do to define the spatial positions of parts of a square, and the particular spatial positions do not matter. In the end, being a square is neither local in requiring elements to occur in a particular place nor literal in requiring green dots or any other specific kind of local stimulus properties. What is crucial to squareness is the spatial relations of the elements, not a concatenation of the pixel values of the elements themselves. Research on human shape perception provides evidence for the primacy of abstract, symbolic representations (Baker et al., 2020a). With their roots in convolution operations, DCNNs excel in leveraging relations of a concrete sort, involving specific local features and color values, but they may lack mechanisms to extract spatial relations, abstracting over the concrete properties of elements (Greff et al., 2020); learning of this sort may require dedicated computational machinery that separates the representation of relations and their arguments (Hummel, 2011).

Some recent work has tested the capabilities of DCNNs to learn visual relations, with particular consideration of their capacities to solve same-different problems. Findings from these investigations indicate that basic DCNNs, as well as some older well-established DCNN architectures (e.g., AlexNet, VGG, LeNet, and GoogLeNet) struggle with same-different tasks, while some newer networks (e.g., ResNets and DenseNets) perform better (Stabinger et al., 2016; Kim et al., 2018; Messina et al., 2021). However, subsequent work by Puebla and Bowers (2021) found that ResNet-50, a 50-layer, enhanced version of earlier ResNets, failed to generalize same-different relations when test images were dissimilar from training images at the pixel level. So far, there is no compelling evidence that deep networks learn relations such that they can apply them to new displays.

In the present work, we aimed to test a variety of relations in visual displays that human perceivers would notice and learn with little effort from a small number of examples, and generalize accurately to new examples. We attempted to replicate and further explore the same-different relation in DCNNs and test two new relations to look at overall characteristics of DCNNs and relational generalization, while using human performance as a comparison.

### 1.2. Plan of the experiments

In Experiment 1, we investigated the learning and generalization of same-different relations in pairs of displayed objects. In Experiment 2, we investigated the relationship of enclosure; each display had a dot that fell either inside or outside of the only closed figure in the display. In Experiment 3, we tested a relationship between color and an object property. Both deep networks and humans were trained and tested in a two-alternative categorization task with displays having two polygons. Whether the display fell into one category or the other depended on whether the polygon with a red dot inside it had a greater or fewer number of sides than the other polygon. For each relation, we trained DCNNs using restricted and unrestricted transfer learning in separate studies. After the completion of training, we tested for generalization to members of the training set withheld during training. We then tested for generalization with new displays that differed in some object characteristics but embodied the same relation that had been the focus of training. In parallel, we also carried out studies with human observers to assess whether the relation in question could be quickly discovered and used for classification and generalization.

## 2. Learning same-different relations

### 2.1. Experiment 1a: Same-different training

We first tested DCNNs' ability to learn same-different classifications. In this task, we placed two novel, closed contours in a single image and tasked the network with learning to produce a “Same” response when the shapes of both contours were the same as each other, and a “Different” response otherwise. The same-different task would be learnable if DCNNs can obtain a feature description of two objects individually within an image and then make a classification decision based on the relation between these two feature descriptions. This differs from standard classification tasks in which the feature descriptions themselves, not the relations between feature descriptions, are pertinent to the network's classification decision.

#### 2.1.1. Method

##### 2.1.1.1. Network

All tests were conducted on AlexNet (Krizhevsky et al., 2012) and ResNet-50 (He et al., 2016), pre-trained on ImageNet (Deng et al., 2009). AlexNet is a high-performing DCNN with relatively few convolutional layers, while ResNet-50 is a much deeper network that represents the current state-of-the-art in feedforward DCNNs.

##### 2.1.1.2. Training data

In each of the experiments presented in this paper, artificial images were generated so that categorization by a DCNN required sensitivity to the relationship being tested. Artificial images, rather than digital images of natural scenes, were used for two reasons. First, it would be difficult to find sufficient number and variety of natural images, and second, it would be difficult or impossible to assess whether classification was based on the relationship of interest, or some other correlated, non-relational cue.

**Figure 1:**
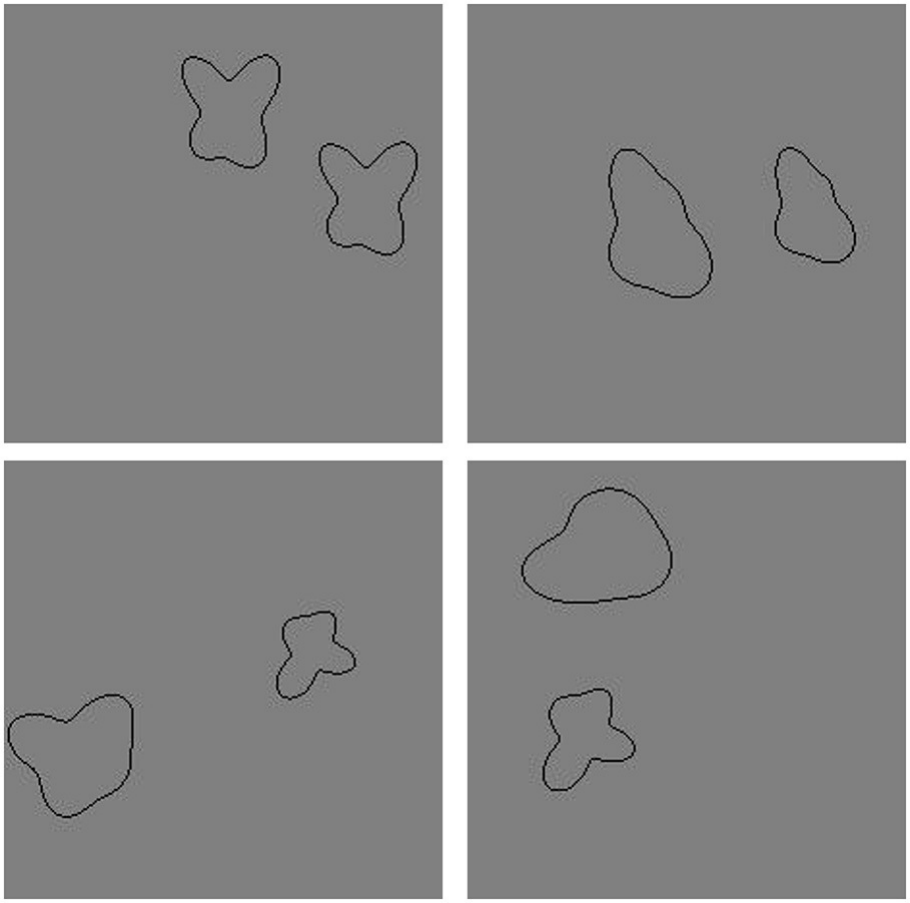
Sample images used during training in Experiment 1a. **(Top)** Two “Same” images. **(Bottom)** Two “Different” images.

We generated 20 novel shapes by moving 10 control points toward or away from the center of a circle, then fitting cubic splines between these control points (see Baker and Kellman, 2018). Training data consisted of images in which one of the 20 shapes appeared twice in the image (“Same” trials) and in which two of the 20 shapes appeared in the image, once each (“Different” trials). In order to prevent overfitting, we placed both shapes in random positions within the image frame with constraints so that the two contours did not overlap and did not touch the image boundary. Each shape was randomly assigned one of 10 sizes, which varied between 20% and 30% of the length of the image frame along the shape's longest dimension. In total, we created 10,000 “Same” and 10,000 “Different” training images. Figure 1 shows some sample “Same” and “Different” images used in training.

##### 2.1.1.3. Training

In order to assess whether DCNNs could learn the same-different relation, we used two different types of transfer learning on an ImageNet-trained AlexNet architecture. In one, we froze all connection weights between convolutional layers in AlexNet, allowing only the last set of connection weights between the penultimate layer and the classification layer to update. We call this restricted transfer learning. Restricted transfer learning tests whether a sensitivity is already latently present from ImageNet training, because the output or decision layer of a network is necessarily based on some weighted combination of the activation of the 4,096 nodes in the penultimate layer. If coding sufficient to detect the presence of two objects of the same shape in a display had evolved in prior training of a DCNN to classify objects, then restricted transfer learning might learn to perform accurately this two-choice discrimination by discovering appropriate combinations of node activations in the penultimate layer.

The second form of transfer learning, unrestricted transfer learning, also begins with a pre-trained network, but allows connection weights at all layers to update during the learning of the new classification task. Unrestricted transfer learning assesses DCNNs' more general capability of obtaining a particular sensitivity, regardless of whether that sensitivity was previously present or not.

We trained with a minibatch size of 32 and an initial learning rate of 1 × 10^−5^. We used 80% of our training data for training and withheld 20% as a validation set. We trained for up to 10 epochs or until error rates on the validation set increased six consecutive times.

For ResNet-50, based on our findings with AlexNet, we used only unrestricted transfer learning. The training data were identical to the data used to train AlexNet. We used a batch size of 50 and an initial learning rate of 1 × 10^−3^. We began by training ResNet-50 for 10 epochs and then did a second training experiment with 70 epochs.

#### 2.1.2. Results

With restricted transfer learning, AlexNet reached criterion after three epochs. Although error rates had increased six consecutive times on the validation set, the network's final classification accuracy showed no evidence of sensitivity to the same-different relation. Performance on the validation set was 54.4%, close to chance performance for the binary classification task, and similar to accuracy levels shown at the end of training. These results suggest that the same-different relation is not something acquired or naturally encoded during training on the ImageNet dataset.

With unrestricted transfer learning, AlexNet reached criterion after 10 epochs. Compared to other transfer learning tasks that do not require a relational comparison (Baker et al., 2020b), learning for the same-different task was both slower and weaker, but the network did eventually improve to 82.2% performance on the validation set, well above chance responding.

After 10 epochs, ResNet-50 did not achieve above-chance classification on the validation set (mean accuracy = 49.7% on the validation set). To assess whether the network simply needed more training iterations to achieve accurate classification, we repeated training with 70 epochs. More extended training produced only a modest improvement in classification accuracy, from 49.7 to 56.0%.

### 2.2. Experiment 1b: Generalization following unrestricted transfer learning

When all connection weights were allowed to update, AlexNet achieved well above chance performance on the same-different task. Our key question here, however, involved what was learned? Did the network learn to attach certain responses to certain images, allowing it to achieve above-chance performance? Or did it come to classify based on detecting sameness or difference between two objects in each display? To test whether the network had learned the abstract “Same” relationship or whether its accurate responses were specific to the shapes we used during training, we generated new images with pairs of shapes that included new shapes qualitatively similar to the shapes used in training, and shapes qualitatively different from those used in training. If the network had come to use the abstract relation, its performance should generalize to new shape pairs.

#### 2.2.1. Method

We used two generalization tests to assess the networks' generalization of the same-different rule. First, we generated 30 new “Same” and 30 new “Different” shapes using the same algorithm previously used to generate the shapes used in training. As in training, the pairs of shapes were given a random size and position in the image frame with constraints to prevent them from overlapping and extending out of the frame.

**Figure 2:**
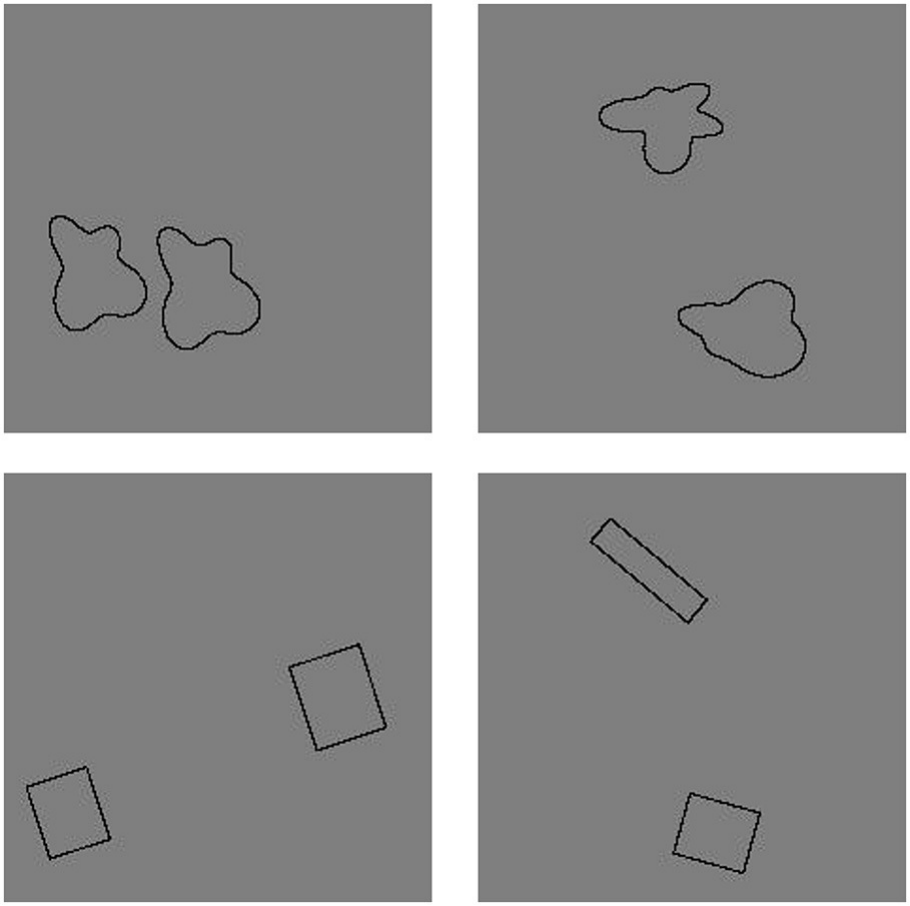
Example generalization test images in Experiment 1b. **(Top)** A “Same” and a “Different” image for the first generalization test. **(Bottom)** A “Same” and a “Different” image from the second generalization test.

We also wanted to test the networks' generalization to the same-different relation using dissimilar shapes. For this test, we used pairs of rectangles. We generated images with two rectangles. The ratio of the minor to principal axis of the rectangles was randomized and varied from 0.08:1 to 1:1. Both rectangles were placed in the image with random size and position. In the “Same” trials, both rectangles in the image had the same aspect ratio and differed only by size and position. In the “Different” trials, the two rectangles differed in aspect ratio as well as by rigid 2D transformations. We generated 30 “Same” and 30 “Different” rectangle pair stimuli. Examples of images from both generalization tests are shown in Figure 2.

We tested both AlexNet and ResNet-50 trained with unrestricted transfer learning on both new sets of stimuli. Because the networks trained with restricted transfer learning never achieved above-chance performance on the validation set, there was no reason to apply the generalization tests to it.

#### 2.2.2. Results

AlexNet's performance was poor in both generalization tests. For the test in which new shapes were generated from the same method as in training, network performance fell from 82% to 58%. For the test with rectangles, performance fell to 50%, with the network classifying all pairs of rectangles as “Same.”

For ResNet, performance was already poor but fell fully to chance on the generalization tests. The network trained with unrestricted transfer learning classified 45% of the new shape stimuli correctly and 50% of the rectangle stimuli correctly.

### 2.3. Experiment 1c: Comparison with humans

The results of our transfer learning experiment on DCNNs suggests they have little ability to use the abstract same-different relation in order to classify images. Humans' registration of same-different relations in perceptual arrays is rapid and automatic (Donderi and Zelnicker, 1969). However, it is possible that our specific paradigm does not elicit perception of sameness/difference in humans. If this were true, then the lack of generalization we saw in DCNNs might not point to a difference in perceptual processing between networks and humans. We tested this by conducting the same experiment we used on DCNNs on human participants.

#### 2.3.1. Method

##### 2.3.1.1. Participants

Six undergraduates (two female, four male, *M*_age_ = 21.0) from Loyola University participated in this experiment as lab researchers. All participants were naive to the purpose of the experiment before completing it.

##### 2.3.1.2. Design

The experiment consisted of a learning phase (150 trials) and two generalization phases (40 trials each). The first generalization phase tested whether classification based on sameness/difference would generalize after learning to new shapes generated in the same way as shapes in the learning phase. The second generalization phase tested pairs of rectangles having the same or different aspect ratios.

##### 2.3.1.3. Stimuli

All stimuli used in the human experiment were taken directly from images used to train or test AlexNet in our DCNN experiment. For the learning phase, we randomly selected 150 (75 same, 75 different) images used during transfer learning. For the generalization tests, we randomly selected 20 same and 20 different images from the same tests used on DCNNs.

##### 2.3.1.4. Procedure

At the beginning of the experiment, participants were told that they would be classifying images into two categories but that they would not be told what defined the two categories. Their task was to use accuracy feedback to discover how to classify images.

During the training phase, participants were shown an image on the screen for 500 ms, after which they were asked whether the previous image belonged to Category 1 or Category 2. After responding, participants were told whether they were correct or incorrect and given the correct classification for the previous image. The image was not shown again during feedback.

Following the training phase, participants completed two generalization tests. They received no feedback during the generalization phases but were told to continue using the same criteria they had adopted during the training phase. In the first generalization test, participants were shown images with the same types of shapes they saw during training, but the actual shapes were different. In the second generalization test, participants were shown images of rectangles with the same or different aspect ratios.

##### 2.3.1.5. Dependent measures and analysis

To assess learning in the learning phase, we separated trials into three 50-trial blocks corresponding to the first, middle, and last third of trials. Because we hypothesized that humans would readily perceive abstract relations such as same vs. different, we predicted that by the second 50-trial block, participants would have learned the rule for categorizing images and should respond correctly for nearly every image.

To assess learning in the testing phases, we simply measured participants' proportion correct and compared their performance on the generalization tests with chance performance and with performance on the final block of the learning phase.

#### 2.3.2. Results

**Figure 3:**
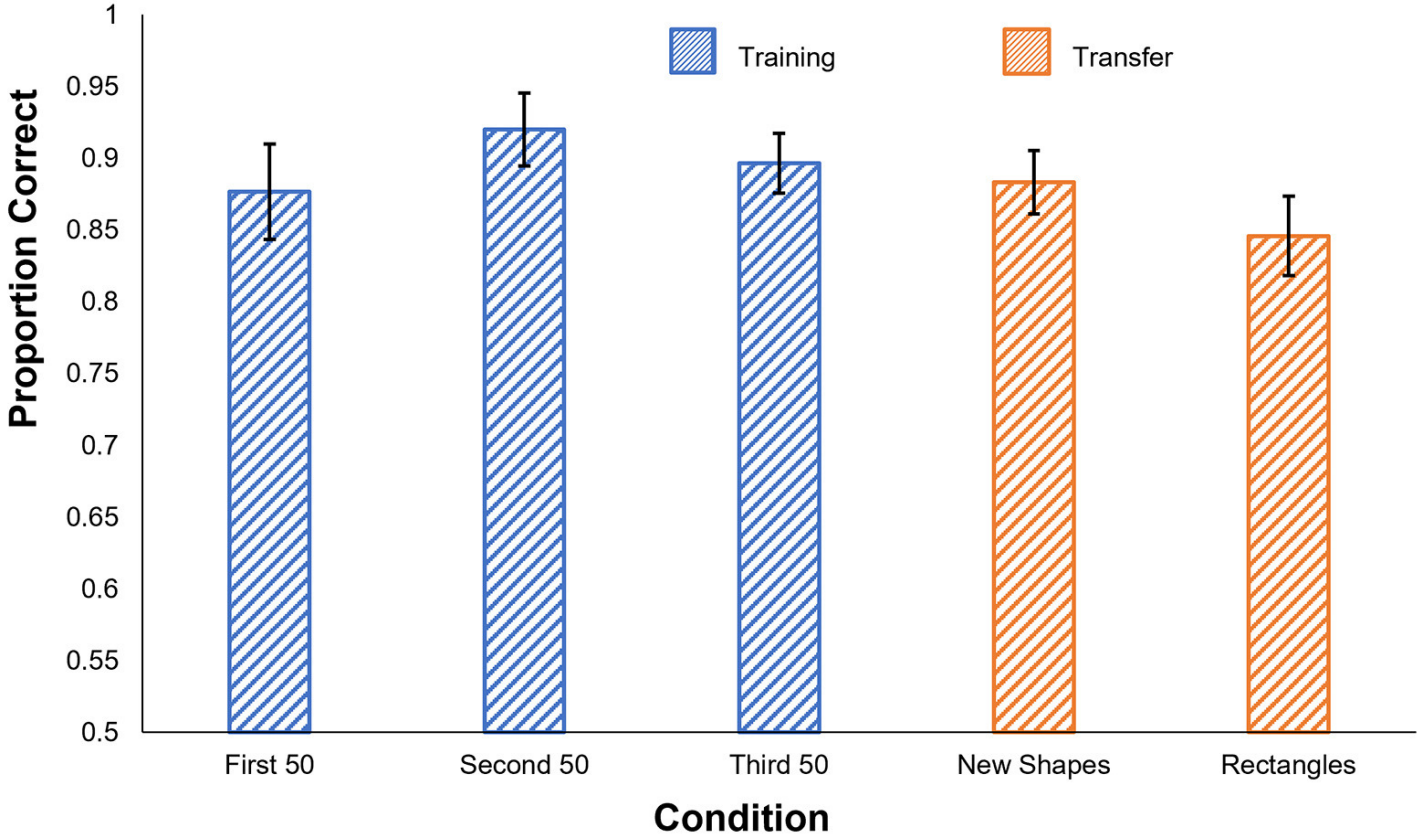
Human results in Experiment 1c. Proportion correct is shown by condition. Blue: performance in the training phase, separated into 50-trial blocks. Orange: performance on the generalization tests. Error bars show ± one standard error of the mean.

The results of the human experiment are shown in Figure 3. Participants performed very well even in the first 50-trial training block and reached ~90% in each of the last two blocks. *t*-tests confirmed that participants performed significantly better than chance in all three training blocks [1st block: *t*_(5)_ = 11.33, *p* < 0.001; 2nd block: *t*_(5)_ = 16.60, *p* < 0.001; 3rd block: *t*_(5)_ = 18.96, *p* < 0.001].

##### 2.3.2.1. Generalization

Participants' accuracy remained high in both generalization tests, significantly exceeding chance levels [New Shapes: *t*_(5)_ = 17.39, *p* < 0.001; Rectangles: *t*_(5)_ = 12.48, *p* < 0.001]. Performance levels also did not significantly differ between the last 50 trials of the training phase and either of the generalization tests [New Shapes: *t*_(5)_ = 1.10, *p* = 0.32; Rectangles: *t*_(5)_ = 1.65, *p* = 0.16].

### 2.4. Discussion, Experiments 1a–c

Research has shown that DCNNs' recognition of objects is primarily driven by texture information, rather than the shape information preferentially used by humans (Baker et al., 2018; Geirhos et al., 2018). Whereas textures and local shape features are composed of locally defined elements, global shape involves relationships among spatially separated parts of object boundaries. Considerable evidence indicates that this more global notion of shape, as opposed to local shape features, is not accessible to DCNNs, even when texture is made non-informative for classification (Baker et al., 2018, 2020b). When texture information is unavailable to DCNNs, they may still achieve above-chance classification accuracy using local contour cues, but not more global features of shape (Baker et al., 2018, 2020b).

We hypothesized that DCNNs' insensitivity to shape may be caused by a more general insensitivity to relational information. To test this idea, we presented the network with a classification task with class type defined by the relation “Same-Different.” With restricted transfer learning, there was no indication that the network could learn this classification. This result is perhaps not surprising, since we did not expect that a DCNN trained for image classification would have sensitivity to global shape. Interestingly, however, with unrestricted transfer, AlexNet did learn to classify the trained shape pairs as same or different (independent of their sizes and positions), but the learning was specific to the trained shapes. Performance was near chance for novel shapes, created through the same generative procedure, and for rectangles. Humans trained with the same shapes showed robust generalization in both cases.

The human visual system is highly flexible, able to represent visual information differently depending on task and stimulus constraints. In numerical cognition research, humans can flexibly switch between perceiving individual objects (Piazza et al., 2011; Cheng et al., 2021), ratios between object groups (He et al., 2009), and objects as a texture field (Burr et al., 2017), depending on stimulus constraints. Similarly, in shape perception, humans can flexibly switch between more local and more global features of a shape (Navon, 1977; Kimchi, 1998; Bell et al., 2007), although the global percept is stronger in many cases. In contrast, DCNNs appear to be much less flexible, making their classifications based only on a small subset of the visual information considered by humans.

The inability of DCNNs to acquire and generalize the same-different relation here is not a finding that arises predictably from prior evidence of the lack of global shape encoding in DCNNs. As mentioned, using unrestricted transfer learning, we did see evidence of acquisition of above-chance performance with the training set. More conceptually, the initial same-different learning task and the first generalization task we posed to the networks could have been accomplished to a high degree of accuracy by use of local shape features without global shape encoding. The notion of same-different can just as well apply to unstructured collections of local features as to global shape. To give one example, in the amoeboid figures, similarities in signs of local curvatures could be informative in determining sameness (in contrast, the rectangles used in the second generalization test may have fewer distinguishing local features; hence all pairs were classified as “Same”). Where available, as in the amoeboid figures, local shape information could have supported the above chance performance on the training set in unrestricted transfer learning. The crucial result regarding relations, however, is that whatever was used to produce correct “Same” and “Different” responses in training showed little or no generalization to new shapes, indicating that whatever was learned, it was not the abstract relation of sameness.

The idea that (somewhat) successful same-different classification observed in training (but not in generalization) was based, not on the relationship same-different, but on the development of sensitivity to the co-occurrence of local features across specific shape pairs aligns with recent work by Puebla and Bowers (2021), who found that DCNNs could only generalize the same-different relation to stimuli that matched training data at a pixel level. The result is impressive, given that the positions and sizes of the shapes in each pair were varied independently, and it underscores the massive capacity for DCNNs to map many different feature combinations onto discrete categories.

The fact that learning did not generalize beyond the trained set, though, as evidenced by the lack of generalization to novel shapes, similarly underscores a key limitation of the operation of these DCNNs. One would expect that, following training, humans could perform this classification on a limitless number of novel shape pairs, provided the shapes themselves were not too complicated or the differences between members of the pairs too subtle. With increased complexity and sufficient training data, a network with this type of architecture would likely be able to learn to successfully classify a larger variety of shape pairs (up to limitations imposed by the vanishing gradient problem), but it would still only be able to classify novel shape pairs to the extent that they resembled pairs in the training data.

In contrast, ResNet-50 never achieved better than near-chance accuracy on the same-different task, even with unrestricted transfer learning and many training epochs. It is puzzling that the deeper network performed worse than AlexNet. Based on AlexNet's poor performance on the generalization tests, it seems likely that whatever rule it was using to perform above chance in training was highly stimulus-specific, not an abstract visual relation. One difference between AlexNet and ResNet is that AlexNet has two fully connected layers between the convolutional layers and the decision layer whereas ResNet has only convolutional layers. These fully connected layers might be important for relating widely spaced features in an image, a process that may be important for the non-abstract comparison furnishing above-chance performance in the training data for AlexNet.

Issues relating to limitations of connectionist networks in capturing or representing abstract relations have been recognized for some time (e.g., Hummel, 2011). The architecture of DCNNs, although more powerful than earlier connectionist approaches, due to both hardware advances (e.g., leveraging GPUs for greater processing power, more memory) as well as algorithmic changes (convolutional layers, skip connections, pooling, etc.), share this same limitation with their ancestors. That said, a more sophisticated network might be able to exhibit some processing of relations, despite these limitations, within a restricted domain. In fact, recent evidence shows that activity in intermediate layers consistent with Weber's Law and sensitivity to the relative sizes of objects, properties that appear to involve simple spatial relations, emerges spontaneously in DCNNs trained for object recognition (Jacob et al., 2021). Our results show, however, that even in this one restricted domain (same-different shape judgments on closed, 2-D contour stimuli), there was little evidence the network could learn to classify based on relational processing outside of the trained set.

It is possible that DCNNs could perform better for other sorts of relational tasks. In Experiments 1a–c, we tested “Same-Different” shape classification performance while allowing for changes in the sizes and positions of the shapes in each comparison pair. Same-different shape classification, while a very intuitive task for people, might be a particularly challenging case for DCNNs. While the task was made easier by not including rotations between the members of a “Same” pair, the network still needed to handle considerable variability both in the shapes themselves and their presentation (i.e., position and size), and to learn to distinguish the features and their relations within a single shape from those between shapes. In Experiments 2 and 3, we consider other relational properties.

## 3. Learning an enclosure relation

In Experiments 2a–b, we investigated a relational property that is perhaps a bit more constrained than abstracting sameness or difference and applying those to novel shapes. We tested the relation of enclosure, specifically, whether a small, locally-identifiable object (a red dot) was inside or outside of a closed contour.

### 3.1. Experiment 2a: Enclosure training

A contour is closed if it has no gaps and its curvature integrates to 360°. In humans, contour closure is a salient cue; it confers perceptual advantages in detection (Kovacs and Julesz, 1993), search (Elder and Zucker, 1993), and recognition tasks (Garrigan, 2012). Experiment 2 specifically aimed to test whether humans and DCNNs can learn to classify images based on an abstract relation between a dot and a closed contour. In one category of images (“Inside”), the dot is within a region is surrounded by a closed contour while in the other category (“Outside”) the dot is outside the region surrounded by the closed contour. Each display had only one closed contour present, along with open contours as noise fragments to eliminate certain possible correlates of enclosure that might otherwise allow DCNNs to perform successfully without detecting the enclosure relation.

#### 3.1.1. Method

##### 3.1.1.1. Network

As in Experiment 1, all tests were conducted on AlexNet and ResNet-50 pre-trained on ImageNet.

##### 3.1.1.2. Training data

For both image categories, we generated a closed contour by moving 10 control points toward or away from the center of a circle and fitting cubic splines between the control points. The shapes were sized so that the greatest distance between two vertical or two horizontal points was between 16.7% and 33.3% of the length of the image frame. The contour was randomly positioned in a 227 × 227 pixel image with the constraint that the whole contour must be within the image frame.

In addition to the closed contour, we added 22 unclosed contour fragments to the image in random positions. The unclosed contour fragments were generated by forming contours in exactly the same way as the closed contour, but selecting only 25–50% of the full contour.

**Figure 4:**
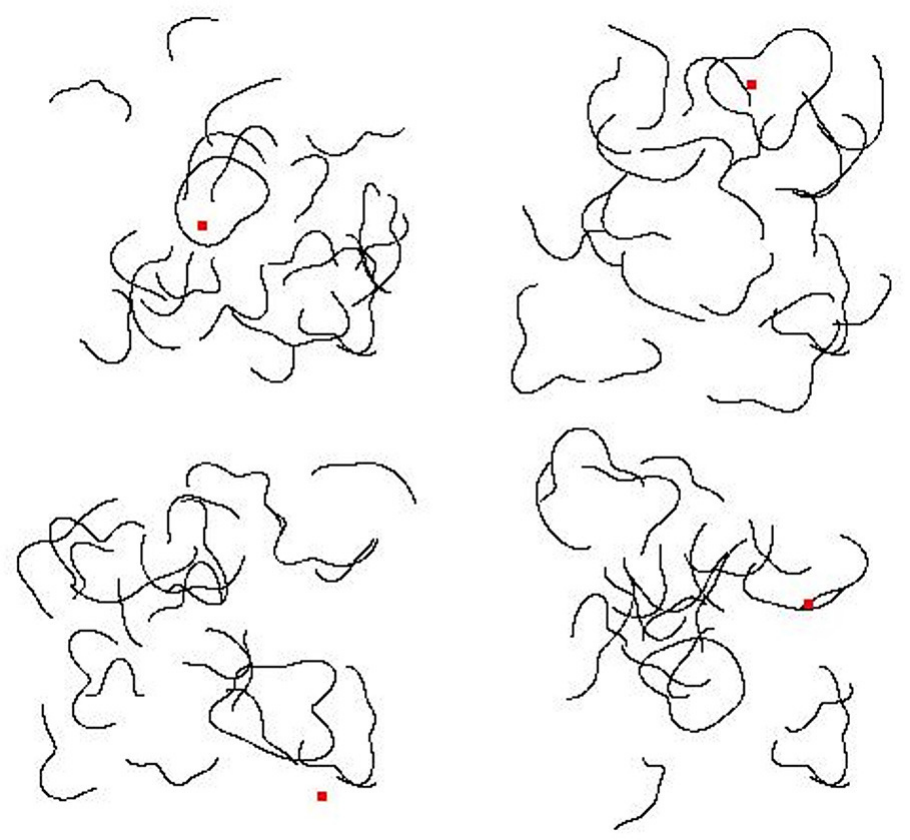
Example training images for Experiment 2a. **(Top)** Two “Inside” images. **(Bottom)** Two “Outside” images. Category membership was determined by the position of the small red square, either inside or outside of a closed contour.

For “Inside” images, we placed a red probe dot in a random position within the closed contour with the constraint that it could not touch the closed contour's border. For “Outside” images, a red probe dot was placed somewhere in the image outside of the region enclosed by the closed contour's border. We constrained the positions of the probe dots in the “Outside” images to be at least 23 pixels away from edges of the full display because these probe positions were unlikely for “Inside” images. We generated 1,000 “Inside” and 1,000 “Outside” images to use as training data for the DCNN. Sample images are shown in Figure 4.

##### 3.1.1.3. Training

As in Experiment 1, we trained AlexNet using both restricted and unrestricted transfer learning. We trained with 90% of our training data, withholding 10% as a validation set. All other training parameters were the same as in Experiment 1. Training concluded after 10 epochs or after the error rate on the validation set increased in six consecutive trials.

Training of ResNet-50 also followed Experiment 1. We trained for 10 epochs using unrestricted transfer learning.

#### 3.1.2. Results

Training with restricted transfer learning ended after eight epochs. The network's accuracy on the validation set was 51.0% after training, around chance levels for a binary classification task. As in Experiment 1, the features learned through ImageNet training do not appear to be usable for the inside/outside task.

Unrestricted transfer learning ended after 10 epochs, with an accuracy of 74.0% on the validation set. These results align with the findings of Experiment 1 and transfer learning in other tasks (Baker et al., 2020b) in that performance was better with unrestricted transfer learning.

Unlike in Experiment 1 where ResNet-50 performed much worse than AlexNet in training, the deeper network performed significantly better in the inside/outside task. Performance reached 99.8% on the validation set after 10 training epochs.

### 3.2. Experiment 2b: Generalization to other enclosure tasks

Had the network learned the abstract enclosure relation? In order to test this, we generated new stimuli in which the inside/outside relation was unchanged, but certain irrelevant image properties differed from the network's training data. The first two generalization tests we conducted tested whether changing contour properties of the closed shape and the open contour fragments would affect the network's classification performance. First, we adjusted a parameter in our generative method for producing shapes to see whether the network generalized. Next, we changed the contours from amoeboids to squares and parts of squares. Our final generalization test evaluated a specific hypothesis that the network's above-chance responding was based on probe dot's proximity to the closed contour boundary, not enclosure of the probe dot. We hypothesized that if this were true, then by making the contour bigger, network performance would fall.

#### 3.2.1. Method

In our first generalization test, we generated shape contours by fitting cubic splines through 16 control points moved away from a circle's boundary rather than the 10 control points used in our training data. Both the closed contour and the contour fragments were generated with 16 control points instead of 10. All other parameters were the same as in the training data. We generated 30 “Inside” and 30 “Outside” images with the new parameter in our generative method.

In our second generalization test, we generated shape contours with squares instead of amoeboid shapes produced by fitting cubic splines through control points. The squares were constrained to be of approximately the same size as the shapes generated in training. As in the training stimuli, open contour fragments were added by randomly selecting 25–50% of square contours that were otherwise matched with the closed contour. We generated 30 “Inside” and 30 “Outside” images with square contours.

**Figure 5:**
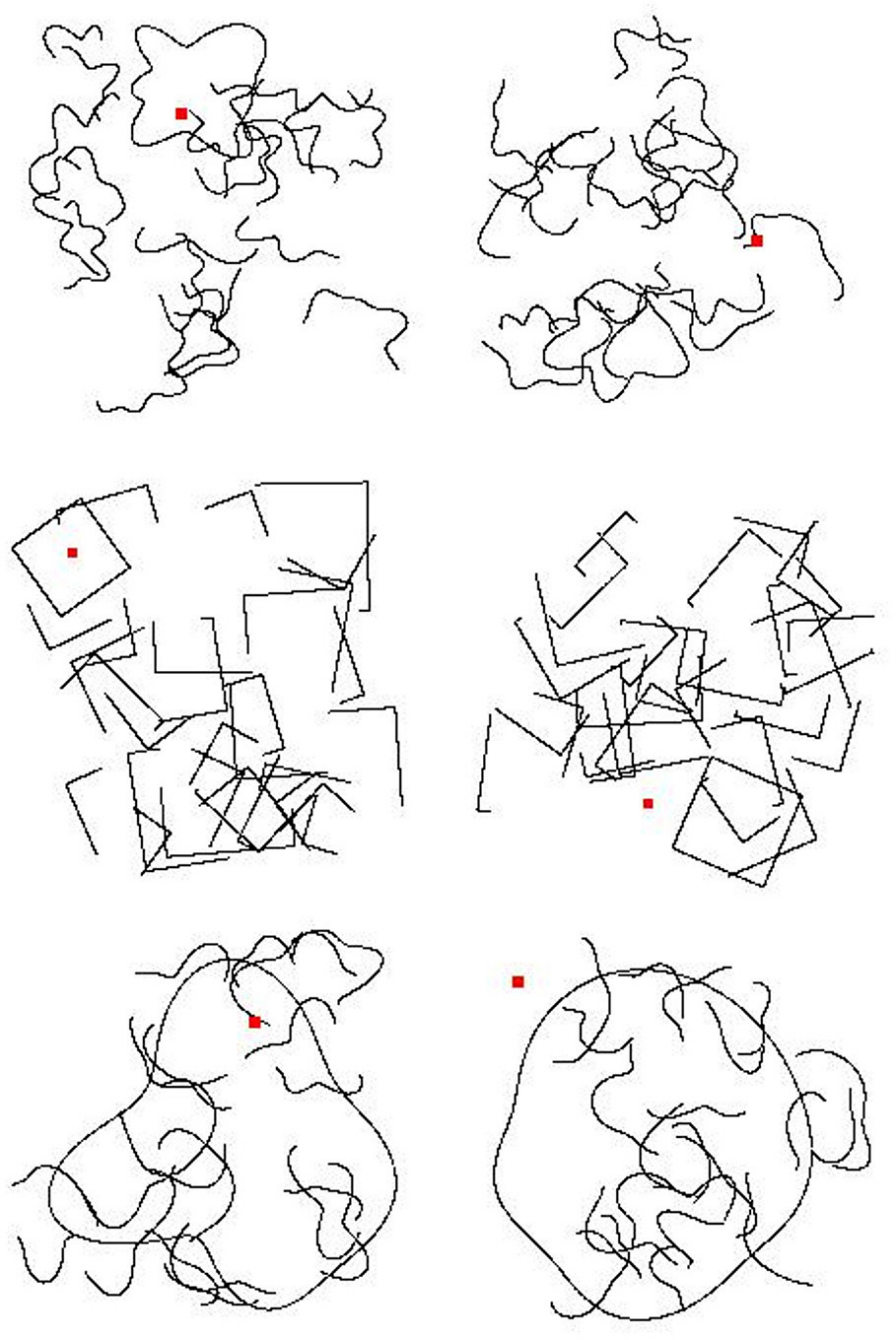
Example generalization test images for Experiment 2b. **(Top)** An “Inside” and an “Outside” image for the first generalization test. **(Middle)** An “Inside” and an “Outside” image from the second generalization test. **(Bottom)** An “Inside” and an “Outside” image for the third generalization test. Category membership was determined by the position of the small, red square, either inside or outside of a closed contour.

In our final generalization test, we kept all parameters the same as in training except that we made the closed shape contour significantly larger to increase the distance between the probe dot and the boundary in “Inside” stimuli. We changed the closed shape's size so that the longest horizontal or vertical distance between any two points on the shape's contour was 80% of the length of one side of the image frame rather than 16.67–33.33% as was used in the training data. Sample images for all three generalization tests are shown in Figure 5.

#### 3.2.2. Results

In all three generalization tests, network performance fell considerably. For the generalization test with 16 control point amoeboids, network performance fell from 74% to 63% for AlexNet and from 99.8% to 76.7% for ResNet-50. For the generalization test with square contours, network performance fell from 74% to 65% for AlexNet and from 99.8% to 59.7% for ResNet-50. For the generalization test with larger contours, network performance fell from 74% to 57% for AlexNet and from 99.8% to 60.0% for ResNet-50.

### 3.3. Experiment 2c: Comparison with humans

Once again, we found little evidence that the DCNN's above-chance performance in the enclosure task was due to apprehension of the abstract inside/outside relation. Instead, DCNNs appear to be using some kind of combination of cues about where in the image the probe dot is positioned (independent of the location of the closed contour) and the probe dot's distance from contours. In Experiment 2c, we tested whether humans, when exposed to the same training displays as networks, learned to use the abstract inside/outside relation and if the use of this relation produced accurate responding on generalization tests.

#### 3.3.1. Method

##### 3.3.1.1. Participants

Six undergraduate (three female, three male, *M*_age_ = 21.0) from Loyola University participated in this experiment as lab researchers. Five of the six participants were the same as in Experiment 1c. All participants were naive to the purpose of the experiment before completing it.

##### 3.3.1.2. Design

Experiment 2c consisted of a learning phase with 150 trials and three generalization phases with 40 trials each. The three generalization phases were the same as those upon which the DCNNs were tested after transfer learning.

##### 3.3.1.3. Stimuli

All stimuli used in the human experiment were taken directly from images used to train or test the DCNNs in Experiment 2a and 2b. We once again selected 150 (75 same and 75 different) images used during the learning phase and 20 same and 20 different images from the generalization tests used on DCNNs.

##### 3.3.1.4. Procedure

The procedure was the same as Experiment 1c. The only thing that differed was the images used during the learning and generalization phases.

#### 3.3.2. Results

**Figure 6:**
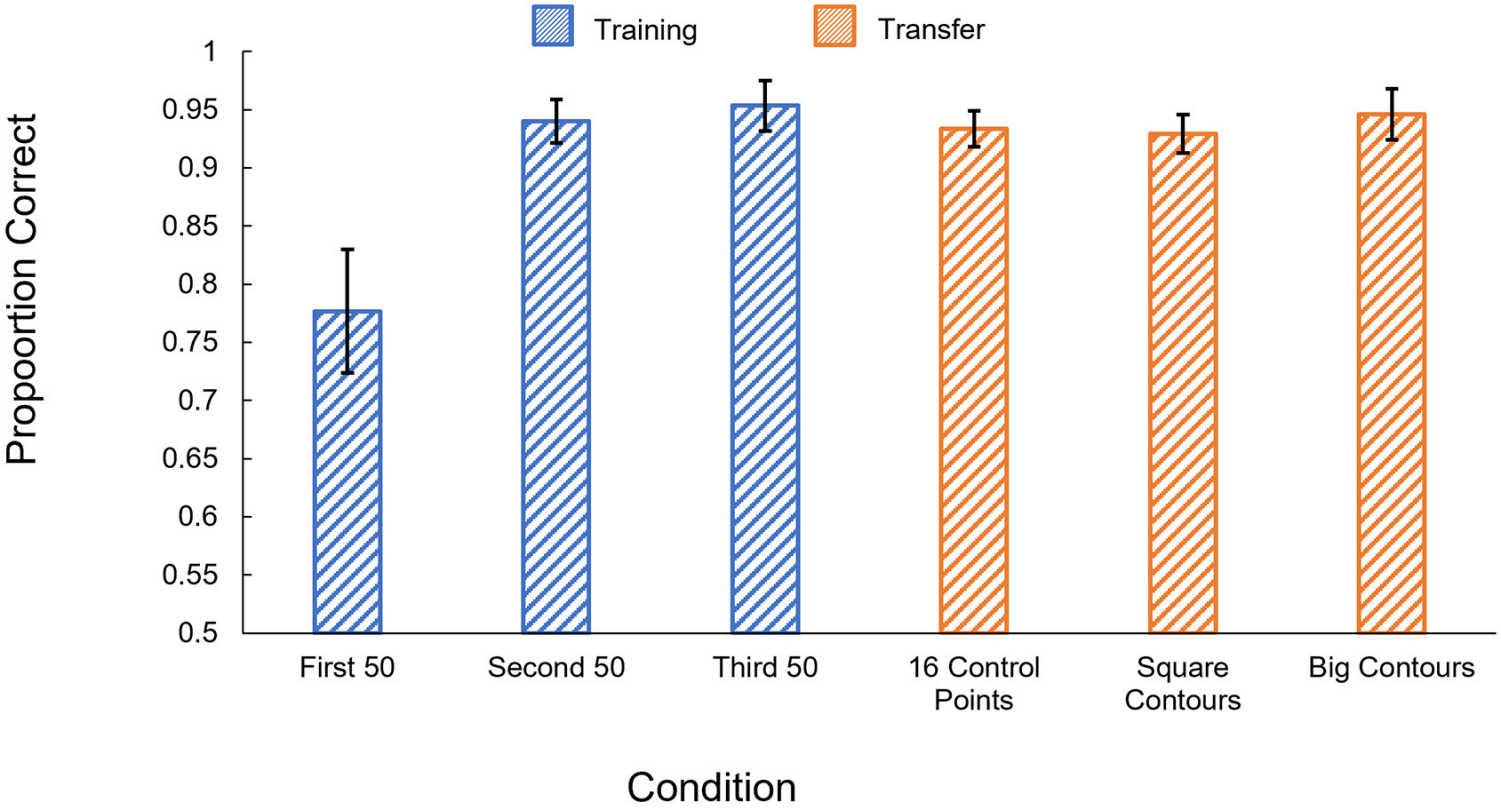
Human results in Experiment 2c. Proportion correct is shown by condition. Blue: performance in the training phase, separated into 50-trial blocks. Orange: performance on the generalization tests. Error bars show ± one standard error of the mean.

The results of Experiment 2c are shown in Figure 6. Participants performed significantly better in the second block of the learning phase trials than the first [*t*_(5)_ = 3.04, *p* = 0.03], but appear to have reached ceiling by the second block and show little improvement from the second block to the third [*t*_(5)_ = 0.54, *p* = 0.61]. Participants performed significantly better than chance in all three training blocks [1st block: *t*_(5)_ = 5.21, *p* = 0.003; 2nd block: *t*_(5)_ = 23.63, *p* < 0.001; 3rd block: *t*_(5)_ = 20.89, *p* < 0.001].

Participants showed robust generalization in all three of our tests, performing significantly better than chance [16 control points: *t*_(5)_ = 28.2, *p* < 0.001; Square contours: *t*_(5)_ = 26.25, *p* < 0.001; Big contours: *t*_(5)_ = 20.44, *p* < 0.001]. Performance also did not significantly differ from performance in the last block of the learning phase for any of the three generalization tests [16 control points: *t*_(5)_ = 0.94, *p* = 0.39; Square contours: *t*_(5)_ = 0.78, *p* = 0.47; Big contours: *t*_(5)_ = 0.27, *p* = 0.80].

### 3.4. Experiment 2a-c discussion

As in Experiment 1, the network was able to perform the classification following unrestricted, but not restricted, transfer learning. Unlike Experiment 1, however, the learning did show some generalization to new conditions, including irregular closed contours generated with a modified procedure (63% and 76.7% for AlexNet and ResNet-50, respectively), and closed rectangles (65% and 59.7%, respectively). We suspected, however, that the network was classifying based on a simpler, more local, relationship—the proximity of the probe dot to a part of any contour in the display. This strategy would naturally account for classification performance reliably above chance, but far from perfect.

**Figure 7:**
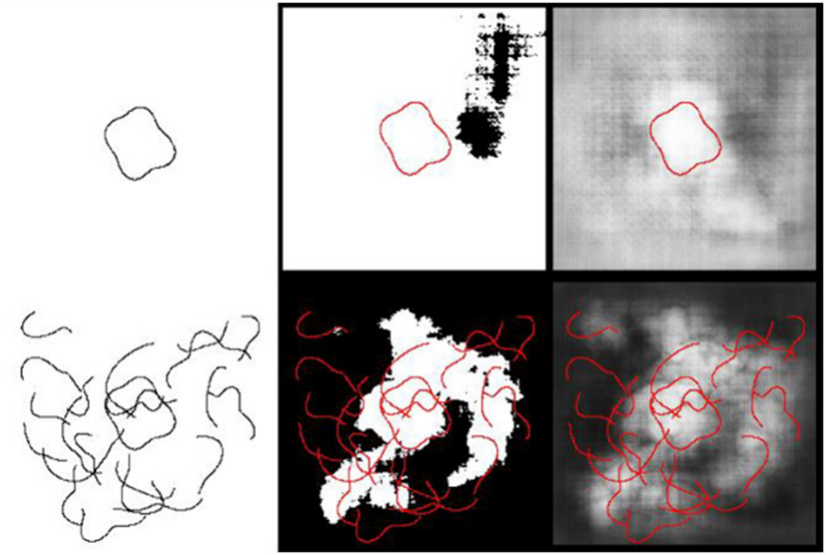
“Inside/Outside” classification of sample images following unrestricted transfer learning in Experiment 2. **(Left column)** Two sample input stimuli are shown, including an isolated closed contour **(left, top)** and the same contour presented among contour fragments, replicating training conditions **(left, bottom)**. In both cases, correct classification of the image was “Inside” only if the probe dot (not shown) was presented within the closed contour. **(Middle column)** Model classification results are shown as binary images indicating the model's classification for various positions of the probe, with the input stimulus superimposed (in red). White indicates probe positions classified as “Inside;” black indicates probe positions classified as “Outside.” **(Right column)** The same results are shown in the right column, but with *p* (inside) indicated by grayscale values (black = 0.0, white = 1.0).

To test this idea, we had the model perform the inside/outside classification with larger closed contour shapes, creating displays with more locations “Inside” the closed contour that were also distant from the contour itself. Consistent with our hypothesis, network training generalized the least in this condition (57 and 60%, for AlexNet and ResNet-50, respectively). We investigated this idea more directly by examining the pattern of correct and incorrect classifications for a specific image. In Figure 7, for two stimuli (one isolated closed contour and the same closed contour presented among open contour fragments), classification performance is analyzed for all possible probe positions. In both cases, for virtually all probe positions inside the closed contour, AlexNet classified that position as “Inside.” The model's behavior for probe positions outside the closed contour, however, provides more insight.

For the isolated contour, most probe positions outside the closed contour were classified as “Inside,” and the errors make little sense for a network sensitive to the actual spatial relationship “Inside.” For example, it is hard to explain why a network that had learned to encode this relationship would correctly classify a probe in the far upper-right as “Outside,” but incorrectly classify probe positions in the three other corners, despite being approximately the same distance from (and not close to) the closed contour. A display with a single, isolated, closed contour, while a useful exploratory tool, is, however, very different from the actual displays used in the training set.

For the closed contour presented among open contour fragments, there was little evidence that proximity of the probe to any contour in the display was driving “Inside” classifications. One might expect errors at probe locations where the contour fragments “almost close,” or where the image is particularly cluttered. However, there is little to suggest this is the case. In Figure 7, middle panel in the bottom row, consider the white region in the central, upper region. Correct classifications of “Inside” are represented by the white region approximately centered in the image, bounded by the red contour. The other white regions represent areas misclassified as “Inside.” The errors observed in these regions cannot be straightforwardly explained by features of the contour fragments nearby them. In fact, other parts of the image appear, by inspection, to have contour fragments that more closely approximate a closed contour (e.g., on the left side, middle).

While it is unclear what strategy the network uses for achieving above chance classifications in the generalization conditions, comparison with human performance strongly indicates that any relational processing by the network is very different from the strategy employed by humans. Humans learned quickly, achieving near ceiling performance by trials 50–100, suggesting that the inside/outside relationship was salient. Further, complete generalization of learning was observed in all cases.

## 4. Learning higher-order relations

### 4.1. Experiment 3a: Network training for higher order relations

In Experiments 1 and 2, we found that humans learn to use perceived abstract relations to categorize images while networks do not. The use of these relations allows human performance to generalize to new stimuli. Networks, although they can learn to classify training stimuli and validation displays similar to the training stimuli, do not extract perceptual relations that allow for generalization of a relation to other kinds of images. Both of the previous experiments tested a simple relation between two image features. For example, in Experiment 1, if the two shapes in the image were the same, the image belonged to the “Same” category. In Experiment 2, if the red dot was within the closed contour, the image belonged to the “Inside” category. These could be called first-order relations because they deal directly with the relation between two properties of an image. A higher order relation would consider a relation between two relations. In Experiment 3, we tested human and DCNNs' ability to classify based on one such higher-order relation.

The images we used in Experiment 3 were displays containing two white polygons on a black background. One of the polygons had a red dot in its center. If the polygon with a red dot had more sides than the polygon without the dot, the image belonged to the “More” category. If the polygon with a red dot had fewer sides than the other, the image belonged to the “Fewer” category. This classification requires the use of a second-order relation because correct responding requires seeing which polygon has more sides, as well as whether that polygon contains the dot.

#### 4.1.1. Method

##### 4.1.1.1. Network

As in Experiments 1 and 2, we trained and tested AlexNet and ResNet-50, pre-trained on the ImageNet database.

##### 4.1.1.2. Training data

**Figure 8:**
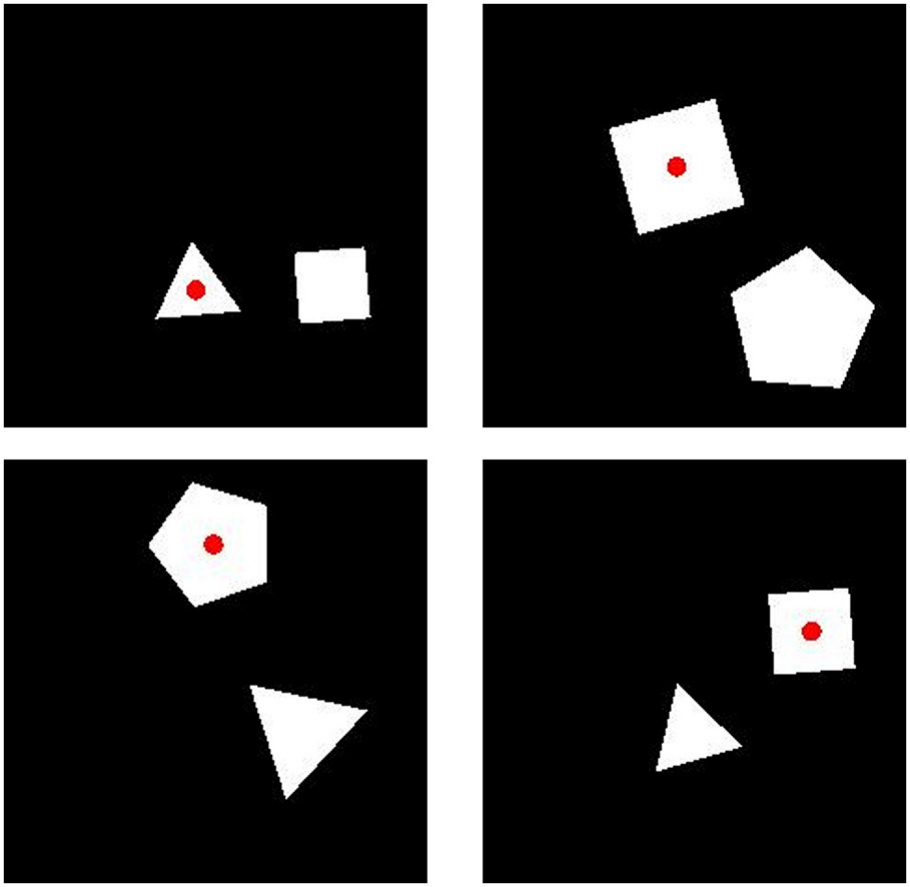
Sample training images for Experiment 3a. **(Top)** Two “Fewer” images. **(Bottom)** Two “More” images. Category membership was determined by the position of the small, red dot, placed on the polygon with either more or fewer sides.

Each image in our training data consisted of two polygons with three to five sides. Images were constrained to always include two polygons with a different number of sides. The size of the image was 227 × 227 pixels. Polygons ranged in length from 22 to 42 pixels and in orientation from 0 to 360°. In each image, we placed a red dot at the center of one of the two polygons. We created 10,000 images in which the red dot was at the center of the polygon with more sides (“More” trials) and 10,000 images in which the red dot was at the center of the polygon with fewer sides (“Fewer” trials). Sample images are shown in Figure 8.

##### 4.1.1.3. Training

As in Experiments 1 and 2, we trained AlexNet using both restricted and unrestricted transfer learning. We trained with 80% of our training data, withholding 20% as a validation set. All other training parameters were the same as in Experiment 1. Training concluded after 10 epochs or after the error rate on the validation set increased in six consecutive trials.

Training on ResNet-50 followed the same procedure as Experiment 2.

#### 4.1.2. Results

Under restricted transfer learning, AlexNet trained to criterion after three epochs and achieved a classification accuracy of 84.4% on the validation set. Under unrestricted transfer learning, AlexNet took eight epochs to train to criterion and achieved a classification accuracy of 99.7% on the validation set, whereas ResNet-50 took 10 epochs to train to a final classification accuracy of 100%.

### 4.2. Experiment 3b: Generalization to other polygons

Despite testing a higher-order relation, network training in both restricted and unrestricted transfer learning was more successful than in either of our previous experiments. The crucial question, however, is whether the network learned response labels for particular concrete features of displays or whether the networks learned the abstract relation between dot location and the relative number of sides of a polygon. In Experiment 3b, we tested this question by generating new test images with polygons with more sides than those to which the network was exposed during training.

#### 4.2.1. Method

**Figure 9:**
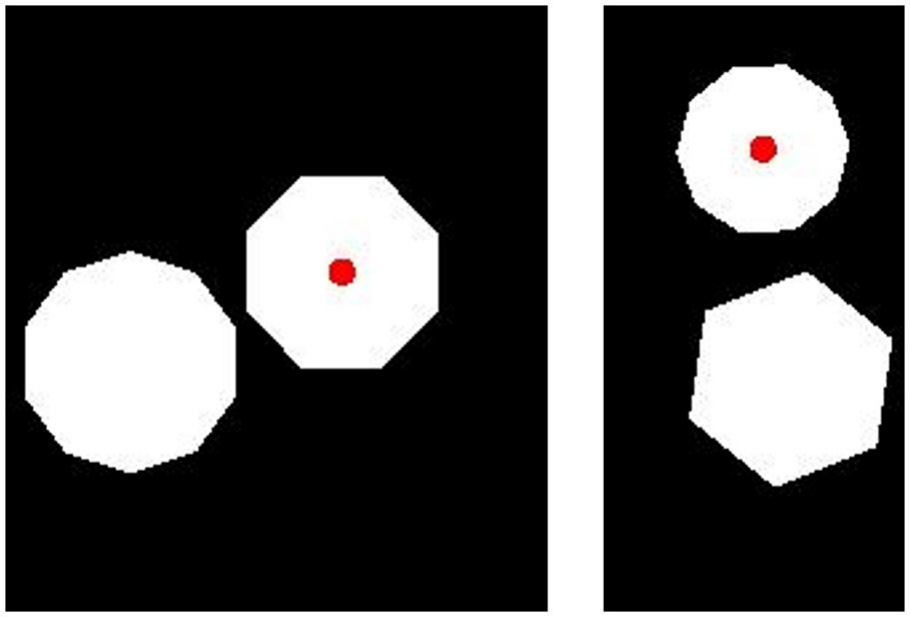
Sample images for the generalization test in Experiment 3b. **(Left)** A “Fewer” image. **(Right)** A “More” image. Category membership was determined by the position of the small, red dot, placed on the polygon with either more or fewer sides.

In our generalization test, we created images with pairs of polygons that had twice as many sides as those present in training images. We replaced all three-sided polygons with six-sided polygons, all four-sided polygons with eight-sided polygons, and all five-sided polygons with ten-sided polygons. In all other respects, the test images were identical to the training images. We produced 50 “More” images in which the dot was placed on the polygon with more sides and 50 “Fewer” images in which the dot was placed on the polygon with fewer sides. Sample test images are shown in Figure 9.

Because AlexNet trained with restricted transfer learning also reached above-chance responding on the validation set, we tested it on the generalization task as well as both networks trained with unrestricted transfer learning.

#### 4.2.2. Results

AlexNet trained with restricted and unrestricted transfer learning had an accuracy of 51% and 50% respectively on the generalization task. ResNet-50 trained with unrestricted transfer learning also had an accuracy of 50% on the generalization task. When we looked into how the networks were responding we found that the network trained with unrestricted transfer learning classified all of the “More” images correctly, but incorrectly classified all of the “Fewer” images as “More.” The network trained with restricted transfer learning did the same apart from classifying one of the 50 “Fewer” images correctly.

### 4.3. Experiment 3c: Comparison with humans

While DCNNs appear able to learn to do the Experiment 3 task in a narrow sense, they showed no generalization whatsoever to other shapes. Performance in the generalization test was even worse for Experiment 3 than Experiments 1 or 2. One reason might be that in Experiment 3, we tested a higher-order perceptual relation than in previous experiments. In Experiment 3c, we tested humans on the same task to see if humans are capable of learning the more abstract relation between stimulus features required for accurate responding in Experiments 3a and 3b.

#### 4.3.1. Method

##### 4.3.1.1. Participants

Twelve participants (seven female, five male, *M*_age_ = 21.0) participated in Experiment 3c. Eight participants were recruited from Loyola University and completed the experiment for course credit and four others were recruited from the University of California, Los Angeles and completed the experiment as volunteers. All participants were naive to the purpose of the experiment before participating.

##### 4.3.1.2. Design

Experiment 3c consisted of a learning phase with 150 trials and a generalization phase with 40 trials.

##### 4.3.1.3. Stimuli

Stimuli from the learning phase were randomly chosen from the network training data (Experiment 3a). Stimuli from the generalization phase were randomly chosen from the network generalization test (Experiment 3b).

##### 4.3.1.4. Procedure

During the learning phase, images were presented in the center of the screen and participants were instructed to classify them into two arbitrary categories (“Category 1” or “Category 2”) with no prior instruction on how to categorize images. Participants were given feedback after each trial and were told to try to discover the correct way of classifying images.

The generalization phase was the same as the learning phase except participants did not receive feedback after they responded.

#### 4.3.2. Results

**Figure 10:**
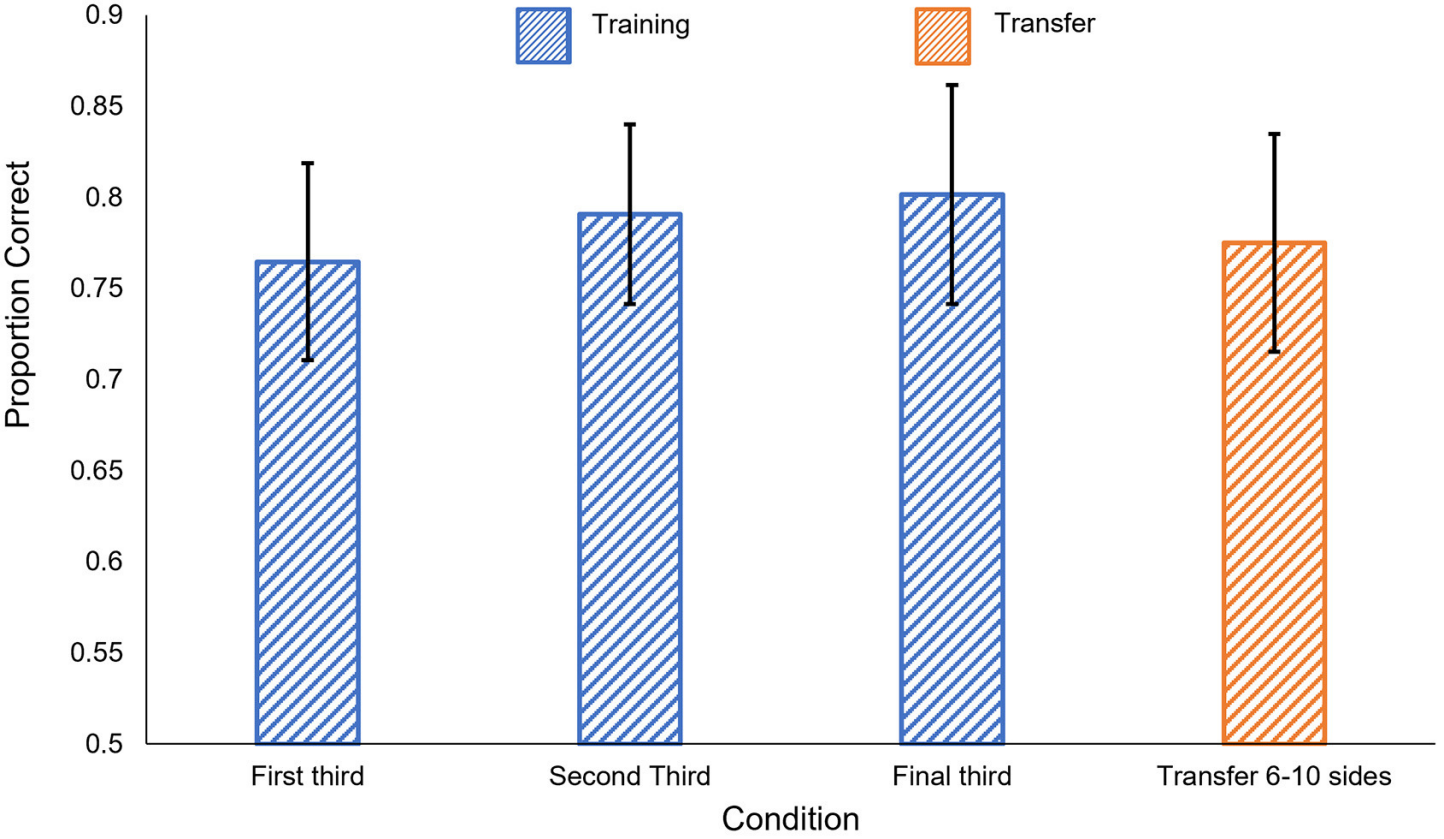
Human results in Experiment 3c. Blue: performance in the training phase, separated into 50-trial blocks. Orange: performance on the generalization tests. Error bars show ± one standard error of the mean.

The results of Experiment 3c are shown in Figure 10. We found no significant difference between the first block of the training phase and either of the two subsequent blocks [*t*_(12)_ < 1.91, *p* > 0.08]. Participants performed significantly better than chance in all three training blocks [1st block: *t*_(12)_ = 4.89, *p* < 0.001; 2nd block: *t*_(12)_ = 5.91, *p* < 0.001; 3rd block: *t*_(12)_ = 5.02, *p* < 0.001].

As in Experiments 1 and 2, participants' learning during the training phase generalized when tested with polygons with more sides. Participants performed significantly better than chance in the generalization task, *t*_(12)_ = 4.59, *p* < 0.001. Performance on the generalization task did not significantly differ from performance on the third block of training, *t*_(12)_ = 0.72, *p* = 0.48.

### 4.4. Experiment 3a–c discussion

As in Experiments 1a and 2a, the networks learned to classify following unrestricted training. AlexNet also learned to classify well above chance performance following restricted transfer learning. Success in the restricted transfer learning case suggests that the features necessary for correct classification of ImageNet exemplars could be repurposed for the current classification task.

Still, neither restricted nor unrestricted transfer learning generalized to a different set of polygons that could be classified by the same rule. Specifically, the network failed to correctly classify polygons with twice as many sides as the training set. Once again, the data indicate that the network did not learn to classify based on a relational property that would generalize to other objects.

The performance of the networks in this study, and to some extent in the earlier studies, raises the interesting question of what was learned by the DCNNs? This is both theoretically interesting in its own right as well as relevant to distinguishing performance that arises from relational encoding from other variables in training displays that may allow powerful networks to exhibit behavior that could naively be interpreted as evidence of relational encoding. In general, it is hard to determine what properties DCNNs use in their responses. Neural networks in general may be characterized as carrying their knowledge in connection weights rather than in explicitly encoded properties. Moreover, the size of contemporary DCNNs allows for a vast array of stimulus variables to influence responses, and even with probing of node responses at various layers, there is no requirement that the properties captured in the network will be intelligible to humans. With regard to the present results, we consider one speculative hypothesis that illustrates how the network achieved some success in training without capturing the abstract relationship in the experiment-defined categorization task. Consider first that the network did learn to classify the polygons in the training set successfully, even following restricted transfer learning, and without, apparently, developing any explicit sensitivity to each polygon's number of sides. If so, it could be that a local feature that distinguishes the polygons, e.g., the internal angles of its vertices, was used in part for the classification task. Sensitivity to a feature like this would not be particularly surprising, given that the ImageNet training set contains many classes of artifacts, including rigid objects, for which the presence of vertices with specific angles might aid in identification. Prior research suggests that DCNNs adeptly capture local shape features (e.g., Baker et al., 2018).

In the initial training, with regular triangles, squares, and pentagons, when the probe was close to a vertex with angle = 108° (regular pentagon), the answer was “yes” (more). When the probe was close to a vertex with angle = 60° (equilateral triangle), the answer was “no” (fewer). A small set of slightly more complicated conjunctive rules allows for classification of the remaining cases without explicitly encoding the relation more-fewer sides. This learning would not, of course, generalize to a different set of polygons with different internal angles.

We expected the more-fewer relationship to be salient to human participants, leading to quick learning and full generalization. This appeared to be the case for the majority of our participants, who classified with >85% accuracy by the end of training and in generalization to polygons with more sides. However, with the added complexity of this classification, relative to Experiments 1 and 2, some participants may have found the perceptual more-fewer judgment too challenging or applied an idiosyncratic strategy. For example, one participant had high performance in training but showed little generalization, a pattern of behavior consistent with learning a complicated conjunctive rule (e.g., red dot in square + triangle = category A, red dot in square + pentagon = category B, etc.) that would have no utility for the different shapes. Another participant had performance in training and generalization testing well above chance, but below the level that would be expected had the more-fewer rule been learned. This participant may have been attempting to classify based on more-fewer sides, but never achieved high performance either because the task was too difficult for them, or perhaps due to poor attention or effort.

## 5. General discussion

The ability to extract abstract visual relations is crucial to many of the most important perceptual processes in human vision, including encoding of shape, arrangement, and structure in scenes, and perception of meaningful properties, such as animacy and causality in events. The notion of abstraction has a range of possible meanings (see Barsalou, 2003, for a useful discussion), but here, we intend a logical sense in which an abstract visual relation is one that involves a predicate that can be detected or represented despite having variable arguments. In perception, this idea is implicit in J.J. Gibson's theorizing about the role of “higher-order variables” in perception (e.g., Gibson, 1979), and more contemporary accounts of abstraction in perception and cognition have emphasized this notion (Marcus, 2001; Hummel, 2011; Kellman and Massey, 2013; Baker et al., 2020a). For present purposes, the impact is that detecting and utilizing abstract stimulus properties requires representations in which the argument is distinct from the relation. For example, a cluster of black pixels in between two clusters of white pixels is a relation, but not necessarily an abstract relation. An alternating ABA pattern of pixels irrespective of the pixel values would be an example of an abstract relation. While deep convolutional neural networks can evolve sensitivity to a vast array of possible “concrete” relations, and these no doubt underwrite their high classification accuracy in particular tasks, it is not clear that they have any access to abstract relations.

In three experiments, we tested DCNNs' ability to learn three abstract visual relations: same-different, inside-outside, and more-fewer. These certainly do not constitute an exhaustive test for all abstract relations, but there are reasons to believe they give valuable insight into DCNNs' general capability of learning abstract relations.

First, each of the three relations we tested depends on a different set of stimulus properties. Same-different depends on the comparison of contours across scale and position, inside/outside depends on the relative positions of the probe dot and a closed contour, and more-fewer depends on the comparison of magnitudes–either a polygon's number of sides or the angular size of its corners. A deficiency in processing any one of these stimulus features might account for insensitivity to one particular abstract relation, but a deficiency in all three relations points to a more general insensitivity to relations of an abstract nature.

Second, the three relations we tested are generally simple and are arguably relevant to systems that use visual information to extract ecologically relevant information from scenes. Experiments 1 and 2 tested what we call first-order relations, or relations between two image properties. Experiment 3 tested a second-order relation between first-order relations. All three are likely to be handled perceptually, given our brief exposure durations and rapid acquisition by most participants from classification feedback alone, and perception of relations in these cases is consistent with other research indicating the perceptual pickup of meaningful relations in scenes and events (Kellman and Massey, 2013; Hafri and Firestone, 2021). These relations all pick up on image features that could be important for object recognition, the task these networks were originally trained to perform. It is therefore reasonable to ask whether relations involving them can be learned in ImageNet-trained DCNNs. These are also the sorts of relations that may be useful in a variety of contexts where meaningful descriptions of objects, spatial layout, and events are to be acquired through visual perception.

The extraction of abstract relations as described here may account for discrepancies previously reported between successful DCNNs and human processing of objects and shape. In human vision, global shape is an abstract encoding in which relations are encoded but the particular sensory elements that act as carriers for relations are often transient, not surviving into more durable representations of objects and shape (Baker and Kellman, 2018). That shape is an abstract, configural notion accounts for the effortless recognition of similarity of shape despite changes in size, orientation, or constituent sensory elements. For example, a relatively small number of rectangles can make an easily recognized giraffe provided that their relative sizes and orientations are appropriate. Even for simple novel shapes, the abstract relations between elements are more important than physical properties of the elements (Baker and Kellman, 2018). The observed incapacity of DCNNs to classify objects based on global shape information likely relates to the general absence of mechanisms that can capture and generalize abstract relations.

We used two training paradigms to assess apprehension of abstract visual relations. In restricted transfer learning, only the weights between the last representational layer and the decision layer were modified by training on a new classification task. In Experiments 1 and 2, we found no improvement in DCNN classification after a full 10 epochs using restricted transfer learning. This suggests that no weighted combination of features learned in ImageNet training could discriminate shapes based on sameness or enclosure. In Experiment 3, AlexNet reached above-chance classification accuracy with restricted transfer learning, indicating that certain features in the image are detected using learned filters from ImageNet training and can be used to discriminate between polygons with more sides and polygons with fewer sides, at least up to 84% accuracy and as long as shapes are within the distribution of polygons on which the network is trained. One possibility is that the network is already sensitive to local features like the angle of corners which can then be associated with distance from the probe dot.

We also tested both AlexNet and ResNet-50 using unrestricted transfer learning, in which all connection weights can be updated. In unrestricted transfer learning, DCNNs can learn new features that might be useful for a specific classification task. In all but one case, unrestricted transfer learning allowed DCNNs to reach performance levels significantly better than chance on the training task itself; however, in the unrestricted transfer learning for Experiment 1, ResNet-50 did not achieve above-chance performance even on the training data.

Most crucial for the questions motivating the present work was whether the networks had achieved training performance in each case by extraction of abstract visual relations or by some other rule that might not be intuitive to humans. We tested this by generating new testing stimuli whose individual features differed from those upon which the networks were originally trained, but could still be classified by the same abstract visual relation. If the abstract relation had been learned, then the network should have classified the new stimuli at the same level of accuracy it had reached on the training data.

Instead, we found that both networks' performance fell off substantially–often to around chance levels–when presented with new stimuli in which the same relations, if detected and used, would have produced perfect performance. The networks' lack of generalization strongly suggests that their improved performance on the training data was due to learning to classify based on a set of stimulus features that were specific to the kinds of images used during training (see Puebla and Bowers, 2021, for convergent evidence). For example, in Experiment 3, they may have learned some conjunctive rule about the kinds of polygons used in training rather than a rule about more or fewer sides that was divorced from the relation's arguments.

The lack of use of abstract visual relations was demonstrated particularly starkly in Experiment 2, where we placed the probe dot at all points within a single image and analyzed the network's pattern of responses. The network's “Inside” responses appeared to depend very little on the features of nearby contours or other relational properties that are easily describable by humans.

This lack of generalization suggests that deep convolutional networks are unable to disentangle relations from the arguments that fill them. In other words, a network might learn to say “Same” when two squares are on the screen, or when two circles are on the screen, but it is doing so in a “conjunctive” manner (Hummel, 2011); the learned relation binds the concrete stimulus features to the response, such that the network will not automatically generalize to say “Same” when two triangles are on the screen. Separating fillers from relations might require symbolic computation, something that does not appear to emerge spontaneously in the training of DCNNs.

We tested human participants with all of the relations presented to DCNNs. In contrast to the networks, humans easily learned all three of the abstract visual relations, often achieving ceiling performance levels in the first 50 training examples. More importantly, human performance was robust in generalization tests with stimuli having features different from than the training data. Across all three experiments, we found no significant difference between human performance on any of the generalization tasks and the last 50 trials in which they were training with feedback.

This difference between humans and networks points to humans' remarkable ability to perceive and use abstract visual relations. It has been argued that even what appear to be simple, basic visual tasks in human visual perception involve abstraction (Kellman and Massey, 2013; Baker and Kellman, 2018). The results presented here show that there are alternative intelligent systems that can be very successful at similar tasks (e.g., image classification) without human-like sensitivity to abstract relations.

Differences between humans and DCNNs also provide a striking example of the flexibility of human visual perception in contrast with the relative inflexibility of processing in deep network architectures. Whereas, humans were able to learn new visual tasks within a few dozen trials of initial exposure, even after tens of thousands of trials, DCNNs were incapable of learning them. Humans' superior flexibility is in one sense unsurprising because, unlike DCNNs, humans are adapted to perform a variety of visual routines that goes far beyond image classification. On the other hand, the case of abstract visual relations is interesting because encoding relations abstractly might crucially underpin our more general flexibility. For example, consider the enclosure relation we examined in Experiment 2. Knowing whether a visual feature is intrinsic to an object or merely correlates with the object can be partly determined by whether it is enclosed by the object's bounding contour. Binding features to objects furnishes a great deal of flexibility in learning about new objects, but it is hard to see how this flexibility and transfer can be accomplished without some representation of abstract notions such as object, boundary, figure vs. ground, etc. Other work suggests that DCNNs do not naturally acquire such representations, such as segmenting the image into figure and ground when learning to classify novel objects (Baker et al., 2018, 2020b).

From the perspective of deep networks, an inability to learn abstract visual relations might be predictive of poor performance on a wide array of visual routines. Processes like segmenting figure from ground (Peterson and Salvagio, 2008), completing an object behind an occluder (Kellman and Shipley, 1991), judging the causality of an event (Michotte, 1954), and representing the shape of objects (Koffka, 1935; Kubovy and Wagemans, 1995; Baker and Kellman, 2018) all depend on access to abstract relations in human vision.

DCNNs may be able to learn appropriate responses in a training set of displays, but without the ability to learn abstract relations, they will perform them in a very different way from humans. An example of this can be seen in comparisons between human and DCNN shape sensitivity. DCNNs do use some shape information (although to a lesser extent than humans), but they use different aspects of shape from humans (Baker et al., 2018, 2020b). These differences can lead to surprising errors in DCNNs, as when an adversarial attack that would be unnoticeable to humans completely changes a network's classification (Szegedy et al., 2013). In the same way, DCNNs might be able to learn responses to other important visual tasks, but without the use of relations. Consequently, we expect that DCNN learning will in general be less robust, and vulnerable to errors that humans would be unlikely to expect (and therefore, in high stakes domains, potentially much more hazardous).

How might DCNNs be enhanced to retain their valuable abilities to learn visual classifications but to also capture abstract visual relations? This is a difficult question to answer because the convolution operators underpinning DCNN operations may be ill suited for the task. Recent ImageNet-trained recurrent (Kubilius et al., 2019) and attention-based (Dosovitskiy et al., 2020) architectures have shown better and more humanlike performance on several tasks, but do not appear to be more sensitive to the global shape of objects (Baker and Elder, 2022). It remains unknown whether a new architecture paired with training data more targeted toward apprehension of visual relations would produce the kind of abstraction observed in humans.

In our view, a more extreme adjustment to these networks might be needed. As argued by Hummel (2011), abstract visual relations might require symbolic processing to separate roles from their fillers. Animal studies have shown that many animals fail to complete same-different tasks that depend on abstract relations (Gentner et al., 2021). However, chimpanzees that are exposed to training with symbolic systems are able to perform well on same-different tasks that chimpanzees with non-symbolic training can not do (Premack, 1983).

Research into symbolic networks has demonstrated that they can represent the spatial relations between parts to build up structural descriptions (Hummel and Stankiewicz, 1996; Hummel, 2001) and to generalize to novel instances of shapes based on their relations (Kellman et al., 1999). It remains unclear how to combine symbolic processing with deep convolutional networks. Some related work on large artificial networks in linguistics (e.g., Vankov and Bowers, 2020; Jiang et al., 2021; Kim and Smolensky, 2021) suggests some strategies for combining extensive associative training with symbolic processing. In vision, capsule networks (Sabour et al., 2018) include some relational coding and have been shown to increase configural sensitivity in uncrowding effects (Doerig et al., 2020). Another recent model adds external memory to a recurrent DCNN to allow for explicit symbolic processing, resulting in rapid abstract rule learning (Webb et al., 2021).

## 6. Conclusion

DCNNs are remarkably accurate image classifiers that, to some degree, mimic human behavior and neurophysiology. These similarities, however, distract from the fact that DCNNs learn very different kinds of visual relations than humans. While humans readily learn relations separable from their arguments, we found no evidence that arguments and their relations are separable in DCNNs. This difference is of fundamental importance. While DCNNs have access to non-abstract relational encoding sufficient for, e.g., human-like performance levels of object recognition, they lack a critical form of representation that supports more general visual perception and reasoning.

Any apparent visual reasoning performed by a conventional DCNN appears to rely on complex mappings among encodings of relatively concrete stimulus properties, rather than any abstract representation of visual information. We believe that this limitation will become more apparent as DCNNs are trained to perform a wider variety of human visual tasks, and may not be overcome with larger, more complex networks. Instead, alternative architectures, possibly ones that explicitly include symbolic computations, and/or modified training regimes, will be needed for DCNNs to apprehend abstract visual relations.

## Data availability statement

The raw data supporting the conclusions of this article will be made available by the authors, without undue reservation.

## Ethics statement

The studies involving human participants were reviewed and approved by IRB, Loyola University Chicago and IRB, UCLA. Written informed consent for participation was not required for this study in accordance with the national legislation and the institutional requirements.

## Author contributions

NB: conceptualization, design, coding, testing participants, simulations, writing, and data analysis. PG: conceptualization, design, coding, and writing. AP: coding, testing participants, and editing. PK: conceptualization, design, and writing. All authors contributed to the article and approved the submitted version.
